# The prevalence of off-label use and supratherapeutic blood levels of outpatient psychotropic medication in suicidal adolescents

**DOI:** 10.3389/fpsyt.2023.1240681

**Published:** 2024-01-17

**Authors:** Isabel Hach, Thomas Bertsch, Patrick Nonell

**Affiliations:** ^1^Department of Education and Science, Klinikum Nürnberg, Paracelsus Medical University Nürnberg, Nürnberg, Germany; ^2^Institute of Clinical Chemistry, Laboratory Medicine and Transfusion Medicine, Klinikum Nürnberg, Paracelsus Medical University Nürnberg, Nürnberg, Germany; ^3^Clinic of Child and Adolescent Psychiatry and Psychotherapy, Klinikum Nürnberg, Paracelsus Medical University Nürnberg, Nürnberg, Germany

**Keywords:** suicidal adolescents, outpatient psychotropic drug use, therapeutic drug monitoring, sex differences, off-label use

## Abstract

**Introduction:**

Adolescents with mental disorders show an increased risk of suicidal phenomena. Vice versa, suicidality is a serious adverse event of psychotropic drug therapy in adolescents. There are only a few new psychotropic agents approved for this young age group. We evaluated the (pre-pandemic) prevalence of off-label use as well as detailed blood concentrations of outpatient psychotropic medication and sex differences in a clinical population of suicidal adolescents.

**Methods:**

The urine presence and serum levels of psychotropic substances of adolescents hospitalized due to their acute suicidality but without a known actual suicide attempt (i.e., no acute intoxication or serious self-injuries) were investigated routinely between 01.03.2017 and 31.01.2018. Urine (*N* = 205) and blood samples (*N* = 193) were taken at the beginning of closed inpatient admission, i.e., the results of the laboratory analysis reflect outpatient drug intake. The serum levels of psychopharmacological medication and OTC medication were measured.

**Results:**

Our sample consists of 231 cases (boys: *N* = 54; girls: *N* = 177, ratio: 1:3.3), aged 12–17 years (average age: 15,4 years). The most prevalent psychiatric diagnoses were depressive episodes (54%) and adjustment disorders (25%), and girls were more often diagnosed with depressive disorders than boys (boy/girl ratio: 1:9.5, *p* < 0.0001). More than half of adolescents (56%) used at least one prescribed psychotropic drug at admission (24.8% ≥ two psychotropic drugs). Off-label use of second-generation antipsychotics was significantly more frequent than off-label use of antidepressants (85% vs. 31%, *p* < 0.01). Adolescents suffering from depressive disorders were significantly more often on-label treated than adolescents with neurotic or stress-related disorders (56% vs. 10%). Female cases with prescribed psychotropic drug use showed significantly more frequent supratherapeutic drug levels than male cases (5% vs. 27%, *p* < 0.05).

**Conclusion:**

Female adolescents may have an increased risk of supratherapeutic blood levels, especially when outpatient prescribed psychotropic drugs are off-label used. Measurement of blood levels of outpatient-prescribed psychotropic drugs could be used to enhance the safety and efficacy of the individual psychopharmacological treatment of adolescent suicidal patients. There is an urgent need for more real-world evidence on the effective treatment of adolescents with psychotropic drugs.

## 1 Introduction

During puberty, emotional fluctuations and even suicidal phenomena (i.e., suicidal thoughts, suicide plans, deliberate self-harm, and attempted suicide) are not uncommon (1, 2). In school samples, 6.5–9% of adolescents reported suicide attempts (i.e., any self-initiated behavior that is designed to lead to death) (1). Suicide is the second leading cause of death among 15–29 year-old young adults by 8.5% (3). Adolescents suffering from mental illness have an increased risk of suicidal phenomena, i.e., compared to adolescents without mental disorders, their risk of suicide is higher by a factor of 3 to 12 (1). Suffering from psychiatric disorders is also a risk factor for non-suicidal self-injury (NSSI) (4–7). In psychiatric adolescent patients, the prevalence rate for repetitive NSSI is found to be up to 50% (5). For comparison, international pooled prevalence rates of NSSI in 10–17 year-old adolescents with and without psychiatric disorders (non-clinical samples, mostly school samples) are approximately 17% (4). Moreover, developing a psychiatric disorder in childhood or adolescence increases the likelihood of continuing to have a psychiatric disorder in adulthood (8, 9). Not only for secondary prevention, early initiation of therapy (e.g., psychotherapy and psychopharmacological therapy) is very important. For adolescents, outpatient treatment is of central relevance, particularly during a pandemic when clinic wards are closed. Psychotropic drugs are often prescribed to adolescents on an outpatient basis, but real-world data on psychopharmacological outpatient treatment of children and adolescents are scarce. Health insurance data can provide prescription rates to adolescents. These prescription rates increased in Germany between 2004 and 2012 (i.e., antipsychotics: 2.3/1000 in 2004 to 3.1/1000 in 2012 in children and adolescents; antidepressants: 4.8/1000 in 2004 to 6.8/1000 in 2012 in the 14- to 17-year-old age group) (10, 11). There are two main problems when analyzing prescription data. Firstly, prescription rates may not accurately reflect actual drug use. Secondly, the indication for the prescription cannot be derived from pure prescription data. In addition, most psychoactive drugs are only approved for use in adults. In Germany, four new antipsychotic or antidepressant drugs are on-label drugs for treatment of adolescents with mental disorders, i.e., aripiprazole (second-generation antipsychotic, SGAP) for therapy of psychotic disorders and manic episodes, fluoxetine (antidepressant drug, AD) against major depressive disorders (MDD), sertraline (AD) against obsessive-compulsive disorders (OCD), and risperidone (SGAP) for short-time therapy (i.e., 6 weeks) against aggressive behavior (12–15). This means that only one new drug per indication is available as on-label medication. Psychiatric patients with the same diagnosis differ, and individual factors of patients require adapted pharmacological profiles. Depending on the predominant symptoms, the choice of psychotropic medication may vary. For example, a sedative antidepressant may be the best drug for a depressed adolescent with severe sleep problems, but there is no new sedative antidepressant approved for this age group. Hence, off-label prescriptions and combinations of psychotropic drugs in children and adolescents are frequent and not limited to psychiatric emergency situations in which they must have their place (16). However, off-label use might be associated with a higher risk of adverse drug reactions, and the risk–benefit balance can be different (17–19). In addition, the evidence base for the use of psychotropic drugs and even new antidepressants in children and adolescents is limited, regardless of whether the drugs are approved or not (10, 11, 18). Acute suicidal behavior in adolescents taking prescribed psychotropic drugs can be both a symptom of the mental disorder and a serious adverse event of the medication (10, 11, 17–19). Both possible causes of suicidality must be investigated and taken into account during treatment. Therapeutic drug monitoring (TDM) is valuable for making psychopharmacological treatment safer and monitoring the success of therapy, particularly in children and adolescents. However, the indication-specific concentration-dose ratios are often missing in this age group (20–22). Different factors may influence the intake of prescribed medication and the measured drug level. For example, being in an emotional crisis (due to a mental disorder) might change the drug intake to a more dangerous use (e.g., overdosing on sedative drugs) or increase non-adherence to existing treatment. Non-adherence to medication is a common problem in adolescent psychiatric patients, especially when adolescents receive a combination of different medications. Missing adherence might contribute to decreased efficacy of psychopharmacologic medication (20). As supratherapeutic drug levels of psychopharmacologic medication might be associated with an increased risk of adverse events (e.g., trouble sleeping, feeling anxious or restless, and feeling sick), they might increase the risk of vulnerable situations (e.g., situations with negative feelings or suicidal thoughts). Also, subtherapeutic drug levels of psychotropic medication might be associated with a risk of poor response, more symptoms of mental disorders, and more suicidal thoughts (20, 23).

In a pre-study for an actual study about risk factors for suicidality and self-injuries in adolescents, we retrospectively evaluated different drug levels of psychopharmacological medication (i.e., TDM) and Over-The-Counter medication (OTC) in blood and urine samples of suicidal adolescent patients. We wanted to find out what types of prescribed psychotropic drugs and OTC medication this population at risk really uses and, particularly, whether these drug levels could provide valuable information for our clinical decision-making process and recommendation for drug therapy.

## 2 Methods

### 2.1 Clinic of child and adolescent psychiatry (Klinikum Nuremberg)

Klinikum Nuremberg is a maximum medical care hospital and one of the largest municipal hospitals in Europe. The Clinic for Child and Adolescent Psychiatry has 53 full-time inpatient and 33 part-time inpatient places, with a ward for children, teenagers, and adolescents or young adults, a ward for the treatment of psychosomatic illnesses, and an emergency ward, as well as two institute outpatient clinics with 3,000 patients per year. The clinic completely serves the Nuremberg region with 1,374,524 inhabitants. Indications for the treatment in the closed emergency ward (six beds) are as follows: endangerment of self and others, acute psychotic symptoms, or accompanied detoxification before withdrawal therapy. Experts categorize patients’ suicidal tendencies on admission (categories: “no,” “low,” “moderate,” “urgent,” or “very urgent” suicidal tendencies). Acutely intoxicated adolescents and/or adolescents in critical conditions have to be treated in acute somatic wards. Between 01.03.2017 and 31.01.2018 (study period), 450 adolescent cases with and without acute suicidality were treated in the closed emergency ward.

### 2.2 Determination of medication in blood and urine samples

The urine presence and serum levels of psychotropic and OTC drugs of adolescents (*N* = 231) hospitalized due to their suicidality but without a known actual suicide attempt (i.e., no acute intoxication or serious self-injuries on admission) were investigated. The samples were routinely captured between 01.03.2017 and 31.01.2018. We included 231 adolescent patients who were clinically judged as acutely suicidal (i.e., “urgent” and “very urgent” suicidal tendencies on admission). Urine and blood samples of acute suicidal adolescents were taken at the beginning of the first closed inpatient admission during the investigation period, i.e., the results of the laboratory analysis reflect outpatient drug intake. We analyzed 205 urine samples (Gas-Chromatography-Mass-Spectrometry GCMS: screening and immunoassay for psychoactive substances: boys: *N* = 40; girls: *N* = 165, boy: girl ratio: 1: 3.3, no detection of aripiprazole) and 193 blood samples (Liquid-Chromatography-Mass-Spectrometry: TDM serum levels: boys: *N* = 51; girls: *N* = 142, boy: girl ratio: 1: 2.8). Twenty-six cases were unable to pass urine at admission. No blood samples could be taken from 42 cases at admission (reasons: refusal or failure). The serum levels and urine presence of prescribed psychopharmacological medication (i.e., antidepressants, antipsychotics, and benzodiazepines) and OTC medication (i.e., aspirin, acetaminophen) were measured: Mirtazapine, sertraline, fluoxetine, risperidone, aripiprazole, pipamperone, quetiapine, diazepam, and lorazepam were measured with liquid chromatography-mass spectrometry in serum; acetaminophen and aspirin with an immunoassay in serum. Ethanol was measured enzymatically in serum.

### 2.3 Ethical considerations and statistical analysis

Laboratory data were anonymized and stored separately from clinical data. The clinical data (e.g., sex, discharge diagnoses, and admission status) of all adolescents (*N* = 450) who were treated in the closed ward during the study period were taken retrospectively from the medical information system of the Nuremberg hospital. Discharge diagnoses were based on referral diagnoses, anamnestic data, and diagnostics performed. Our approach was in accordance with the IRB protocol that was approved by the local institutional review board (IRB 2022_002). The data were merged via case numbers. Data were analyzed using MS Excel. Frequencies, percentage distributions, and means were used as the descriptive analysis methods. The comparison of categorical variables was analyzed using the chi-square test (*χ*^2^). Statistical analysis was carried out anonymously. The significance level was defined as *p* < 0.05.

### 2.4 Reference ranges

We examined if serum levels of new-generation antidepressants and antipsychotics approved in Germany for adolescents or in clinical studies tested and frequently prescribed were in the therapeutic range (12–15, 24–27). We used the following therapeutic reference ranges: aripiprazole 100–350 ng/mL, quetiapine: 100–500 ng/mL, risperidone (i.e., risperidone plus 9-hydroxy-risperidone): 20–60 ng/mL, fluoxetine (i.e., fluoxetine plus N-Desmethylfluoxetine): 120–500 ng/mL; mirtazapine: 30–80 ng/L, sertraline: 10–150 ng/mL, pipamperone: 56.0–180.5 ng/mL (23, 27). In Germany, pipamperone is indicated for sleeping disorders and psychomotor agitation (i.e., symptoms and behavioral problems, no diagnoses). We could not judge whether or not pipamperone use was on-label.

## 3 Results

### 3.1 Sample and main diagnoses

Our sample consists of 231 acutely suicidal adolescent cases (boys: *N* = 54; girls: *N* = 177, boy/girl ratio: 1: 3,3), aged 12–17 years (average: 15.4 years). More than half of cases suffered from (recurrent) moderate or severe depressive episodes (main diagnoses ICD-10: F32.1, F32.2, F33.1, F33.2, 57%). Girls were significantly more often diagnosed with MDD than boys (male cases *N* = 13 vs. female cases: *N* = 111, ratio: 1:9.5, *p* < 0.0001). Approximately a quarter of cases (24%) showed adjustment disorders as the main diagnosis (ICD-10 F43.2) (Table 1). More than a third of cases (*N* = 85) had injuries or superficial lesions of the skin at admission (e.g., forearm 34%; hip, thigh, knee, and lower leg: 26%). Most cases were admitted to the hospital as emergency patients (*N* = 206, 89%). In adolescent cases with acute suicidality, the proportion of girls was significantly higher than in non-acute suicidal cases (76.6% vs. 64.8%). They suffered significantly more often from depressive disorders (53.7% vs. 24.2%) and significantly less often from (hyperkinetic) conduct disorders (5.2% vs. 17.8%), personality disorders (1.7% vs. 7.8%), and mental disorders due to psychoactive substance use than not acutely suicidal cases (1.7% vs. 6.8%).

**Table 1:** Main diagnoses of adolescent cases with and without acute suicidality (*N* = 450) and prevalence of psychotropic drug treatment (according to blood concentrations) of adolescent cases with acute suicidality.

	Sample		Blood samples of acute suicidal adolescents (TDM) *N* = 193 (100%)		
	Adolescent cases without acute suicidality (*N* = 219) No blood samples	Adolescent cases with acute suicidality *N* = 231 (100%)	Blood samples of cases without psychotropic drug use (*N* = 109, 56.5%)	Blood samples of cases with psychotropic drug use (*N* = 84, 43.5%)	
				Off-label treated cases *N* (%)	On-label* treated cases *N* (%)
Female	142 (64.8%)	177 (76.6%)	80 (56.3% of female cases)	35 (56%)	27 (44%)
Male	77 (35.1%)	54 (23.4%)	29 (56.9% of male cases)	13 (57%)	9 (43%)
Mental disorders (main diagnoses)*					
Mental disorders due to psychoactive substance use F1	15 (6.8%)	4 (1.7%)	2	2	0
Schizophrenia F20.0, schizoaffective disorders F25.1	6 (2.7%)	7 (3%)	0	0	5 (100%)
Major depression F3	53 (24.2%)	124 (53.7%)	61	18 (44%)	23 (56%)
Moderate depressive episode F32.1		61 (27%)			
Severe depressive episode (SDE) F32.2		35 (16%)			
SDE with psychotic symptoms F32.3		6 (2.6%)			
Recurrent depressive episode (RDD): moderate episode F33.1 severe depressive F33.2		22 (10%)			
Neurotic and stress-related disorders F4	84 (38.3%)	79 (33.8%)	40	27 (90%)	3 (10%)
Adjustment disorders F43.2		57 (24.7%)			
Posttraumatic stress disorder F43.1		16 (6.9%)			
Obsessive compulsive disorder F42.2		4 (1.7%)			
Personality disorders F6	17 (7.8%)	4 (1.7%)	0	2 (100%)	0
Emotionally unstable personality disorder F60.31		3 (1.3%)			
Disorders of psychological development F8	4 (1.8%)				
Behavioral and emotional disorders F 9	39 (17.8%)	13 (5.2%)	6	4 (100%)	0

*According to ICD-10 Chapter V Mental and behavioral disorders main blocks (categories) F1- F9.

### 3.2 Gas-chromatography-mass-spectrometry and immunoassay-screening

We did a quantitative and qualitative analysis of 205 urine samples (Table 2). Theobromine (i.e., a product of the metabolism of caffeine and cacao) and tetrahydrocannabinol (THC, detected by immunoassay) were the most often found psychoactive substances in urine (theobromine: *N* = 153, THC: *N* = 35), followed by prescribed antipsychotics and/or antihistamines (*N* = 87, quetiapine: *N* = 23; promethazine: *N* = 21; chlorprothixene: *N* = 18; pipamperone: *N* = 17; diphenhydramine: *N* = 3; phenotiazine: *N* = 5) and antidepressant agents (total: *N* = 67, mostly SSRIs, e.g., fluoxetine: *N* = 27, sertraline and citalopram *N* = 15 each). Detection of aripiprazole was not possible in urine. Approximately a quarter of cases (*N* = 66, 28%) showed no psychotropic or analgesic medicaments in their urine. Ibuprofen was found in 27 samples, and aspirin and acetaminophen were found only in single cases.

**Table 2:** Found psychotropic substances with GCMS screening (*N* = 205).

Antipsychotics/Antihistamines (*N* = 95)	Antidepressants (*N* = 66)	Anxiolytics/ Sedatives (*N* = 23)	Others (*N* = 17)	Number of psychoactive drugs found together (*N* = 106)
Alimemazine: *N* = 2	Amitriptylin: *N* = 1	Alprazolam: *N* = 1	Ambroxol: *N* = 1	1 psychoactive drug: *N* = 64
Chlorprothixene: *N* = 17	Citalopram: *N* = 15	Diazepam: *N* = 1	Alpha-Tocopherole: *N* = 1	2 psychoactive drugs: *N* = 34
Diphenhydramin: *N* = 3	Fluoxetine: *N* = 27	Lorazepam: *N* = 21	Amphetamines: *N* = 5	3 psychoactive drugs: *N* = 12
Methotrimeprazine: *N* = 2	Mirtazapine: *N* = 3		Carbamazepine; *N* = 1	4 psychoactive drugs: *N* = 5
Olanzapin: *N* = 2	Opipramol: *N* = 1	Analgesics OTC (*N* = 34)	Hydrocortison: *N* = 3	5 psychoactive drugs: *N* = 1
Phenothiazine: *N* = 5	Sertraline: *N* = 15	Diclofenac: *N* = 2	Oxcarbazepine: *N* = 1	
Pipamperoen: *N* = 21	Venlafaxine: *N* = 4	Ibuprofen: *N* = 27	Spartein: *N* = 1	
Promethazine: *N* = 19		Acetaminophen: *N* = 3	THC: *N* = 35	No substances: *N* = 66
Quetiapine: *N* = 24		Aspirin: *N* = 1	Theobromine: *N* = 123	

More than half of our sample (56.8%) used at least one psychotropic drug at admission, and 51 cases (24.8%) showed ≥2 psychoactive (prescribed) drugs in GCMS. Combinations of antipsychotic drugs with antidepressant drugs were frequent. Sedative antipsychotics (e.g., chlorprothixene, promethazine, pipamperone) were used by 69 female and 19 male adolescents (male–female ratio: 1:3.3).

### 3.3 Therapeutic drug monitoring

We analyzed 193 blood samples. Prescribed psychoactive drugs were detected in 84 cases (43%). Male and female cases took psychoactive medication with almost the same frequency (boys: 43% vs. girls: 44%). Cases suffering from schizophrenia or schizoaffective disorders were always treated with on-label psychoactive medication. Approximately 40% of adolescent cases with MDD and 43% of cases with neurotic or stress-related disorders received psychotropic medication (Table 1). Psychotropic medication in adolescents suffering from MDD was mostly used on-label (56%), whereas only 10% of adolescents diagnosed with neurotic or stress-related disorders received on-label medication (*p* < 0.001). Female cases showed significantly (*p* < 0.0246) more often supratherapeutic drug levels than male cases (Table 2).

#### 3.3.1 Therapeutic drug monitoring of antipsychotics (SGADs and pipamperone)

**Figure 1:**
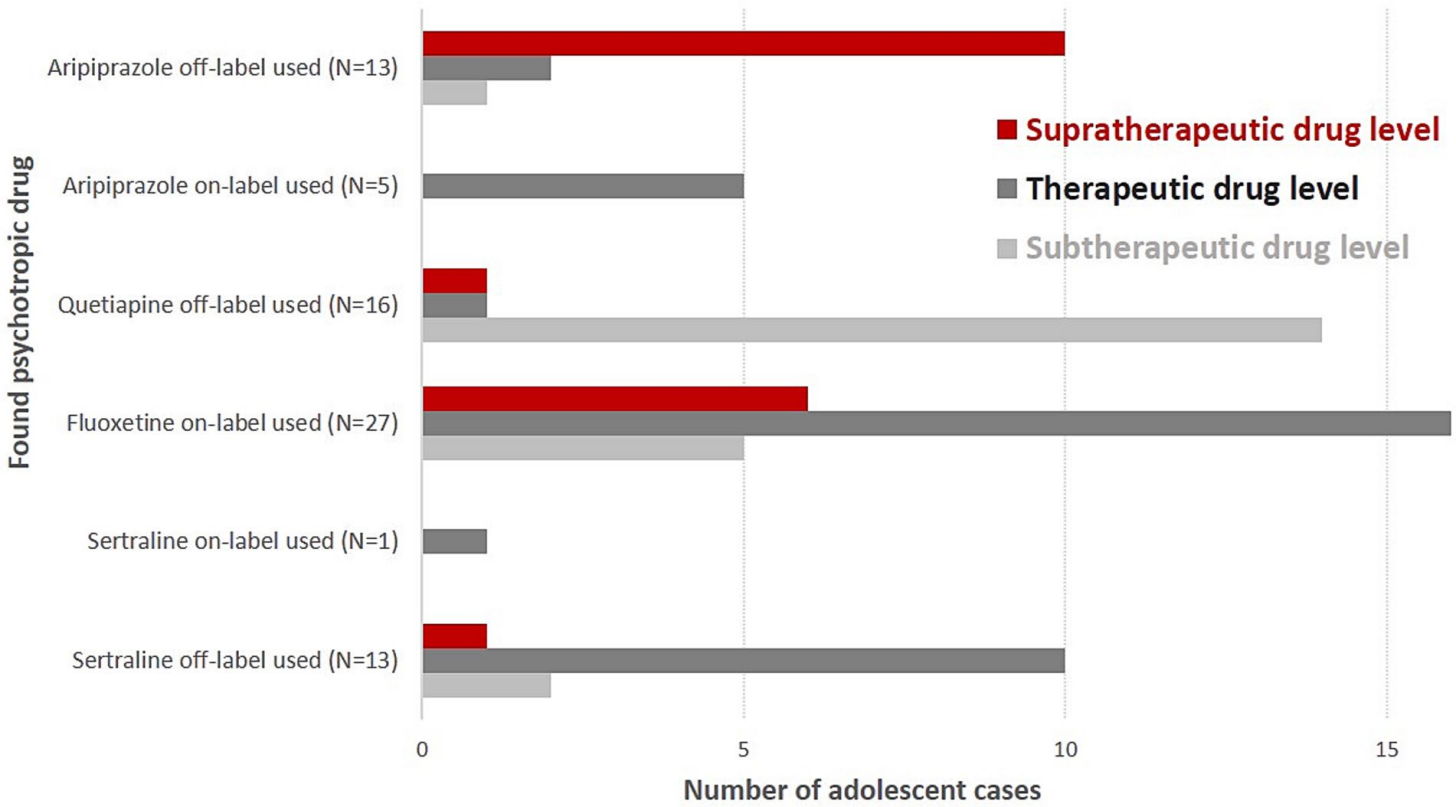
Supra-, sub-, and therapeutic drug levels of on- and off-label-used psychotropic drugs in suicidal adolescent cases.

Aripiprazole was the most often found antipsychotic drug in blood samples (*N* = 18; only female cases, off-label use: *N* = 13, supratherapeutic drug levels: *N* = 10), followed by quetiapine (*N* = 16; 5 male cases, 11 female cases, ratio: 1:2.2, off-label use: *N* = 16, subtherapeutic drug levels: *N* = 14) (Table 2 and Figure 1). Five adolescent cases used risperidone (one male and four female cases, subtherapeutic drug levels: *N* = 3, therapeutic drug levels: *N* = 2).

All five cases diagnosed with schizoaffective disorders, depressive type (*N* = 4), or schizophrenia (*N* = 1) received aripiprazole (i.e., 100% on-label use). We found 10 supratherapeutic aripiprazole serum levels in female cases suffering from adjustment disorders (*N* = 9 between 383 ng/mL- 569 ng/mL, N-dehydroaripiprazole between 70 ng/mL-89 ng/mL, i.e., between 13 and 21% of aripiprazole serum levels). The highest serum level of aripiprazole at admission was 1910 ng/mL (dehydro-aripiprazole: 416.0 ng/mL) in a 14-year-old girl with adjustment disorder (main diagnosis) and influenza, attention deficit disorder, and attachment disorder as secondary diagnoses. Her co-medication was quetiapine (13 ng/mL, N-desalkylquetiapine, 165 ng/mL), diazepam, and chlorprothixene (both positive in GCMS). THC was also found in her urine. Two cases with aripirazole use showed MDD (F32.1, F32.2), and one girl was diagnosed with unsocialized conduct disorder (F91.1). Most quetiapine serum levels were subtherapeutic (i.e., <100 ng/mL, 87%). One supratherapeutic level (945 ng/mL, N-desalkylquetiapine, 153.0 ng/mL) was found in a 17-year-old girl with co-medication of fluoxetine (serum level: 302 ng/mL). She had the main diagnosis of MDD, and “multiple superficial injuries of the forearm” was her secondary diagnosis. Quetiapine was frequently found in combination with antidepressants (four times with fluoxetine and sertraline each, two times with pipamperone, and once with risperidone). One case received risperidone as monotherapy. Two cases with risperidone medication also used fluoxetine and benzodiazepines. Fifteen patients showed pipamperone serum levels at admission (sub- and therapeutic levels: *N* = 14; one supratherapeutic level: 350 ng/mL). Pipamperone was combined with other low-potential APs (e.g., chlorprothixene and promethazine: three cases) and/or with fluoxetine (five cases). Five cases received pipamperone as monotherapy (Table 3).

**Table 3:** Results of therapeutic drug monitoring (TDM) at admission (*N* = 193 blood samples).

Substances assessed Therapeutic reference range (ng/ml) Detection limit (ng/ml) Positive samples	Aripiprazole 100–350 <20 *N* = 18	Quetiapine 100–500 <10 *N* = 15	Risperidone 20–60 <2 *N* = 5	Pipamperone 56–180 <6.0 *N* = 15	Fluoxetine 120–500 <14 *N* = 27	Sertraline 10–150 <2 *N* = 14	Mirtazapine 30–80 <2 *N* = 3	Nordazepam 120–800 <15 *N* = 3	Lorazepam 30–100 <4 *N* = 16
Concentrations found (ng/ml) (in bold: within therapeutic reference range)	91, **173**, **181**, **211**, **240**, **251**, **282**, 327, 383, 388, 412, 438, 497, 528, 553, 561, 569, 1910	10, 11, 13, 13, 17, 17, 19, 26, 29, 41, 41, 44, 46, **184**, 945	3, 11, 15, **44**, **45**	9, 14,15, 15, 30, 41, 42, 44, **58**, **72**, **97**, **106**, **108**, **132**, 350	32, 45, 50, 60, 109, **138**, **183**, **188**, **189**, **197**, **199**, **200**, **206**, **211**, **234**, **238**, **302**, **303**, **333**, **365**, **438**, 526, 558, 565, 599, 676, 851	2, 9, **22**, **23**, **28**, **34**, **35**, **52**, **67**, **80**, **99**, **139**, **140**, 162	11,11,14	21,71,76	7, 7, 7, 7, 7, 7, 8, 9, 12, 14, 16, 19, 21, 21, 23, **32**
Mean (Median) (ng/ml)	444 (385.5)	97 (26)	29 (15)	76 (44)	296 (211)	64 (43.5)	12 (11)	56 (71)	14 (10.5)
Supratherapeutic levels	*N* = 10	*N* = 1	*N* = 0	*N* = 1	*N* = 6	*N* = 1	*N* = 0	*N* = 0	*N* = 0
Therapeutic levels	*N* = 7	*N* = 1	*N* = 2	*N* = 6	*N* = 16	*N* = 11	*N* = 0	*N* = 0	*N* = 1
Subtherapeutic levels	*N* = 1	*N* = 13	*N* = 2	*N* = 8	*N* = 5	*N* = 2	*N* = 3	*N* = 3	*N* = 15
Main diagnoses According to ICD-10 Chapter V Mental and behavioral disorders (main blocks F1- F9)	F2: *N* = 5 F3: *N* = 3 F4: *N* = 10	F3: *N* = 7 F4: *N* = 5 F6: *N* = 3	F3: *N* = 4 F4: *N* = 1	F1: *N* = 1 F3:*N* = 9 F4:*N* = 3 F9:*N* = 2	F3: *N* = 24 F4: *N* = 3	F1: *N* = 1 F2:*N* = 2 F3: *N* = 5 F4: *N* = 4 F6: *N* = 2	F3: *N* = 1 F4: *N* = 2	F3: *N* = 2 F4: *N* = 1	F2: *N* = 2 F3:*N* = 7 F4: *N* = 6 F9: *N* = 1
Females (F) Males (M)	F: *N* = 18 M: *N* = 0	F: *N* = 13 M: *N* = 2	F: *N* = 4 M: *N* = 1	F:*N* = 13 M:*N* = 2	F:*N* = 24 M:*N* = 3	F: *N* = 9 M:*N* = 5	F:*N* = 1 M: *N* = 2	F: *N* = 2 M: *N* = 1	F: *N* = 9 M:*N* = 7

#### 3.3.2 Therapeutic drug monitoring of antidepressants and benzodiazepines

Fluoxetine was the most often detected antidepressant agent in our blood samples (*N* = 27, 4 male cases, 23 female cases, ratio: 1: 5.75; subtherapeutic drug level: *N* = 5, supratherapeutic drug level: *N* = 6), followed by sertraline (*N* = 14, 5 male cases, 9 female cases, ratio: 1: 1.8; therapeutic drug level: *N* = 11; Figure 1). Fluoxetine was always used within indication and often as monotherapy (74%). Almost 50% of cases with fluoxetine medication (*N* = 12) showed superficial injuries (mostly of the forearm) as secondary diagnoses. Comorbid anxiety disorders were found in seven cases. One female case diagnosed with recurrent severe depressive episode and fluoxetine monotherapy showed the highest supratherapeutic level of fluoxetine in our sample (fluoxetine 665 ng/mL, D-Fluoxetine 186 ng/mL, sum: 851 ng/mL). Twelve cases with fluoxetine use had as secondary diagnoses “superficial injuries” and seven cases showed comorbidity, mostly anxiety disorders. According to its indication (i.e., obsessive-compulsive disorder; not depressive disorders), sertraline was mostly used off-label. All but one of our sertraline users showed subtherapeutic (*N* = 2) or therapeutic (*N* = 11) drug levels. Mirtazapine serum levels that were found were always subtherapeutic once it was in combination with sertraline. Sixteen cases showed lorazepam drug use (7–32 ng/mL), and three cases had nordazepam (i.e., the first metabolite of diazepam) levels (21, 71, 76 ng/mL). Diazepam, temazepam, and oxazepam were not found in our blood samples (measured by the LCMS method).

### 3.4 Comparison of the use of off-label aripiprazole and off-label sertraline

Adolescent cases using on-label aripiprazole always showed aripiprazole serum levels in the therapeutic reference range. Adolescent cases using off-label aripiprazole very often had supratherapeutic drug levels (*N* = 10, 83%). Off-label sertraline users showed supratherapeutic drug levels significantly less frequent than off-label aripiprazole users (*N* = 1 vs. *N* = 10, *p* < 0.0001).

## 4 Discussion

We started our study with the goal of finding out what types of psychoactive drugs prescribed to adolescent outpatients were sex- and diagnosis-specific and which blood concentrations adolescents show. The main strength of our study is that our data reflect actual psychotropic drug consumption at admission. Secondly, we wanted to answer the question of whether TDM at the beginning of an inpatient treatment could be an instrument for improving (psychopharmacological) treatment of suicidal adolescents at risk.

### 4.1 Sample and diagnoses

Especially in mid-adolescence, girls suffer more often from suicidality and show NSSI than boys. The three times higher proportion of female cases in our sample may reflect this fact as well as the fact that adolescent cases with acute suicidality were significantly more likely to be girls than adolescent cases without acute suicidality (28, 29). We assume (classification of NSSI is not possible using ICD 10) that the diagnoses of superficial injuries/lesions, for example, at the forearm, are cutting or scratching and intentional self-inflicted injuries which are also more prevalent in girls than in boys (5–7). Self-harm and depression frequently occur together in adolescent psychiatric emergencies (16). Girls are also more frequently affected by depression and anxiety symptoms than boys (28). Female cases in our sample showed significantly more often MDD than males. Approximately 20% fulfilled the diagnostic criteria of recurrent and/or severe depression (ICD-10: F32.3, F33.1, and F33.2). Recurrency and severity of depression at a young age are associated with poorer outcomes (8, 9). In accordance with Dobson et al. (30), the acutely suicidal adolescents in our sample suffered more often from depressive disorders and less often showed externalizing disorders (e.g., hyperkinetic conduct disorders) or autism spectrum disorders than non-acutely suicidal adolescents. We found that approximately 25% of suicidal adolescents were diagnosed with adjustment disorders. Our results are in line with a Swiss study that described that approximately 20% of adolescents presented to an emergency department with reactions to stress and adjustment disorders (29).

### 4.2 Psychotropic drugs and substances found by GCMS and immunoassay screening

Suicidal adolescents of our sample used different psychoactive substances (caffeine, THC, and prescribed and OTC medication) at admission. Caffeine consumption might be inversely associated with depressive symptoms in adults (31), but the presence of Theobromine is not able to indicate such a relation. The prevalence of cannabis consumption in adolescents in Germany is approximately 8% (32, 33). Not surprisingly, in our clinical sample, we found THC twice as much. Cannabis has sedative effects. Sedation may be one of the reasons adolescents with depressive symptoms consume cannabis. There are pharmacokinetic (e.g., with fluoxetine) and pharmacodynamic (especially with other sedatives) interactions between THC and prescribed psychotropic drugs (34). Hence, the simultaneous use of cannabis and psychotropic drugs may increase the risk of ADRs. Moreover, cannabis consumption increases the risk of developing depressive disorders and suicidality as well as triggering psychotic episodes. Cannabis has harmful effects on memory function, especially in young age groups. Cannabis consumption is dose-dependently associated with depressive disorders and suicidality in vulnerable persons (32). There were only four cases of substance use disorders (SUD) diagnosed as the main diagnoses. The most often found psychotropic drugs were antipsychotics. Abbas et al. described an increase in antipsychotic drug prescriptions in German adolescents and suggested a critical discussion on prescribing practices (10). We found indicators for polypharmacy (i.e., concurrent treatment with >2 psychotropic drugs). If we assume that the time criterion for polypharmacy was met (i.e. overlapping concurrent treatment with different psychoactive substances for a duration of at least 7 days (Egbert et al.), 30 days (Chen et al.) or 60 days (Toteja et al.)), up to a quarter of our cases may have received polypharmacy in the outpatient setting (19, 35, 36). One rationale for prescribing different psychotropic drugs at the same time might be the need to address several areas of symptomatology without producing too many side effects. Temporary treatment of exacerbation of mental disorders might be another reason for using polypharmacy, particularly in inpatient settings. However, our sample consisted of outpatients with consumption of different psychotropic drugs at the same time; thus, if prescribing doctors had the intention to prevent the worsening or the exacerbation of mental illness, they probably failed, as most of our cases were emergency admissions. Polypharmacy increases non-adherence. Polypharmacy is also a risk factor for ADRs. The most serious ADR in adolescents with psychotropic medication is suicidality (2, 18). However, in a closely monitored clinical setting, the risk of serious ADRs did not significantly differ between adolescent patients using psychotropics off-label and on-label (19).

### 4.3 Therapeutic drug monitoring of psychotropic medication

#### 4.3.1 General findings

The type of frequently prescribed antipsychotic medication (i.e., low-potency antipsychotics) and subtherapeutic levels of high-potency SGAP quetiapine suggest that the intended effect of prescribing physicians might have preferentially been sedation and/or reduction of self-injurious-behavior-and-not-therapy-of-existing psychotic or manic episode. Subtherapeutic drug serum levels might be both a result that was unintended by prescribing physicians that might indicate non-adherence or an intended result to avoid adverse events (21). For MDD and anxiety disorders, SGAPs are commonly prescribed in adolescents and adults (10, 17). Antipsychotics in our sample were often used off-label. The finding of off-label use is in accordance with other studies, but in our sample with outpatient prescriptions, in the case of aripiprazole, it is mostly due to indication (78%), whereas, e.g., Egberts et al. described off-label use of antipsychotics in 63.2% due to age (19). The reasons only female adolescents received aripiprazole and why aripiprazole was not prescribed to adolescents suffering from MDD with psychotic symptoms remains unknown. Probably, it was by accident or due to the small sample size of patients with psychotic disorders. Other studies found aripiprazole prescriptions more often in male adolescents than in girls [e.g., (37)]. Adverse events that occur in male adolescents in a higher frequency could be a reason for non-prescription in our sample, e.g., low serum prolactin levels are more often found in male than in female adolescents (48–53% vs. 25–28%) (12). Fluoxetine was always used on-label and often within the therapeutic reference range (13, 21). Sertraline was frequently used off-label as well, but almost all suicidal adolescent cases showed sertraline serum levels in the therapeutic reference range (14, 21, 23).

#### 4.3.2 Therapeutic drug monitoring of SGAPs

All five female cases with on-label prescribed aripiprazole showed aripiprazole blood concentrations within the therapeutic reference range. Cases with neurotic and stress-related disorders more often received off-label prescribed SGAPs than cases with MDD. Ten female cases with adjustment disorders (i.e., off-label use) showed aripiprazole concentrations in blood above the therapeutic reference range. Tolerability decreases above that upper limit (12). Therapeutic improvement is less likely (20, 27). Clear recommendations on how to treat adjustment disorders are missing; moreover, knowledge of pharmacological treatment is very limited (38). All reference ranges are indication-specific. Aripiprazole is approved only for the therapy of psychotic disorders. The evidence for its efficacy in other mental disorders is limited (12). We found no strong indicators for dangerous use (quantitative or qualitative) of prescribed psychotropic drugs, as there were only two singular cases with remarkably supratherapeutic SGAP drug levels and without obvious co-medication. Both cases with highly elevated supratherapeutic levels may have taken higher dosages of their SGAP immediately before admission. They showed higher serum levels of aripiprazole and quetiapine than of their related metabolites. As in steady state conditions, the active metabolite dehydro-aripiprazole is approximately 40% of aripiprazole available in plasma; we assume that the very much elevated supratherapeutic drug level is not only a result of continuous (prescribed) overdosage. An additional intentional or unintentional singular overdosage is more probable (12). The diagnosed virus infection could have played a role as well. The Cmax and AUC of N-desalkylquetiapine are approximately 20% higher in adolescents than in adults. However, everything considered, the observed N-desalkylquetiapine level of 416 ng/mL seems too high even for an adolescent suffering from a virus infection. The serum concentration of quetiapine might be increased when it is combined, as in our case, with fluoxetine. Hence, the supratherapeutic serum level of quetiapine may be a result of those pharmacokinetic aspects (24).

Aripiprazole is not effective against depression and has no relevant sedating effects. Moreover, aripiprazole did not prevent the recurrence of depressive episodes in adults and had no effects on relapse prevention of manic episodes in adolescents (12). Acute or chronic depressive symptoms may not have been the target indication in our sample. However, Aripiprazole may have implications on the dopaminergic and serotonergic transmission and reduce NSSI. The effectiveness of SGAPs in reducing NSSI was shown in some clinical studies (5–7). Reducing NSSIs and/or reducing symptoms of borderline personality disorders (clinical prevalence of borderline personality disorders is 11% in adolescents and up to 76% in emergency departments) might have been a therapeutic impact of aripiprazole and quetiapine prescribed by physicians (40).

#### 4.3.3 Therapeutic drug monitoring of pipamperone

Pipamperone is a frequently prescribed popular low-potential AP in Germany that is indicated for the therapy of behavioral problems. We can only judge based on main or secondary diagnoses if psychotropic drugs were on- or off-label prescribed. Outpatient use of pipamperone might have been on-label if it was prescribed after consideration of the benefit–risk-ratio and check of indication. However, from an evidence-based perspective, as randomized controlled clinical trials with depressed adolescent patients are lacking, the use of pipamperone should be limited (39). The same applies to other low-potential APs that were detected with GCMS (e.g., chlorprotixene: due to the absence of randomized controlled studies, it is not recommended for children and adolescents).

#### 4.3.4 Therapeutic drug monitoring of antidepressants

Depressive disorders were the most common main diagnosis in our sample. In contrast to antipsychotics, antidepressants were frequently used on-label in our sample. Antidepressants, especially fluoxetine, might be a good option to treat depressive symptoms in adolescents (40). However, there is the general question of whether antidepressant drug therapy in adolescents is indicated, as the evidence of pharmacological treatment is often missing (18). Fluoxetine was often found in cases suffering from MDD and acute superficial injuries or psychiatric comorbidity. Antidepressants may reduce NSSI. In addition to their neurobiological influence, the successful treatment of depressive disorders with SSRIs may increase the capacity of patients to reduce their self-injurious behavior. More indications could have led to more prescriptions as one antidepressant might treat different mental disorders at the same time.

Given that an antidepressant drug at a therapeutic level reduces depressive symptoms better than an antidepressant agent at a subtherapeutic (i.e., low therapeutic response) or supratherapeutic level, there is much potential for improvement as five of our depressed adolescent cases showed subtherapeutic levels of fluoxetine and six cases had supratherapeutic levels (27). Sertraline serum levels were mostly in the therapeutic range as well. Fluoxetine and sertraline differ slightly in their chemical structure, but their pharmacodynamic properties are similar (13, 14). All SSRIs show quite similar pharmacological characteristics, whereas SGADs differ. Our results suggest that off-label-used aripiprazole necessitates more intensive monitoring of blood levels and adjustment within the therapeutic range than off-label-used sertraline. For the treatment of OCD, higher doses of sertraline might be required compared to major depressive disorder (MDD) (14). However, Tini et al. found effective serum concentration levels between approximately 66 and 76 ng/mL for the successful treatment of adolescents with OCD (23). It is noteworthy that subtherapeutic levels of mirtazapine also suggest that it probably might not have been prescribed due to its antidepressant effect but due to its sedating effect (26). One case received mirtazapine in combination with fluoxetine. Combinations of SSRIs with α2-autoreceptor antagonists are potent treatment options in adult non-responders of antidepressant monotherapy (41). The number of patients with mirtazapine medication in our sample is limited, and further interpretations are not possible.

#### 4.3.5 Therapeutic drug monitoring of benzodiazepines

We could not find indicators for frequent outpatient use of benzodiazepines. The often-prescribed lorazepam is a short-acting and rapidly cleared benzodiazepine, and for this reason, we have probably not been able to find it frequently in blood samples. Benzodiazepine use is increasing with age, e.g., middle-aged and older females in the United States show prevalence rates of any benzodiazepine use between 7.1% (35–50 years of age) and 10.8% (65-80y), whereas only 3.8% of 18–35 years old women used benzodiazepines (42). It is noteworthy that sedation seemed to be an important target of antipsychotic medication in our sample. Maybe the depressed and anxious, sedated, and/or over-dosed female adolescents of our sample will be middle-aged benzodiazepine-(dependent) women of the future.

### 4.4 Recommendations for suicidal adolescents with and without psychotropic use

**Figure 2:**
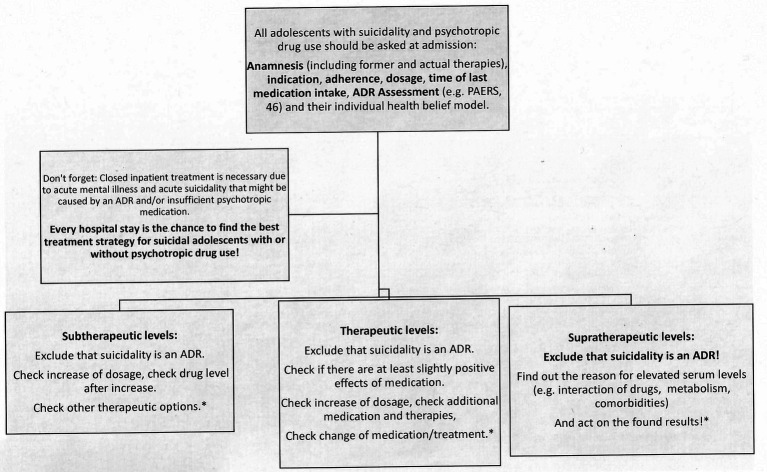
Treatment strategy for suicidal adolescents with prescribed psychotropic medication in relation to observed drug levels. *In accordance with guidelines and treatment recommendations (20, 21, 41, 42, 44, 45, 51, 52)].

We used quite an unusual way and investigated routine drug levels at the beginning of inpatient treatment, unaware of the prescribed drugs and circumstances of medication. Please note that the adolescents in our sample are a population at risk due to their mental illness, suicidality, emergency admissions, consumption of cannabis, and (polypharmacy of) psychotropic medication. Although the treatment of depression is a highly relevant problem, studies on the quality of treatment are limited (43). Most adolescents were already in contact with psychiatric outpatient treatment, as they showed prescribed psychotropic medication in their blood and/or urine. Hospital admission and acute illness, respectively, might indicate non-response to prescribed medication or ADRs. Although Egberts et al. described that off-label use of psychotropic drugs in clinical settings was not associated with an increased SAE rate in youths, special attention should be paid to adolescents with off-label use and abnormal blood levels in our sample (19). Our adolescents are “only” suicidal, and they usually come to the hospital before they have tried to admit suicide. Every inpatient treatment is an opportunity to care for all adolescents suffering from mental disorders with all the multi-professional competence that inpatient treatment can offer, i.e., to find the best individual guideline-based therapy for each patient [e.g., (38, 40, 43, 44)]. Adolescents who are not taking medication should be advised about the appropriate pharmacological treatment options. For adolescents who are already taking medication, the results of the laboratory tests (TDM, GCMS) could serve as a basis for discussion. First, a decision should be made as to whether a psychopharmacological treatment is (still) indicated at all. In case of continuing the prescribed psychotropic medication, dose adjustments should be made in the hospital, using TDM. Whenever possible, deprescribing to reduce polypharmacy and to react to supratherapeutic levels of (off-label) medication should be an important part of hospital care (27). In the case of depressed adolescents with prescribed antidepressant medication and subtherapeutic levels, after anamnesis, proof of adherence, and dosage check, we recommend an increase of dosage and serum level control after 7 days (TDM). The exclusion of an ADR could be carried out with the Paediatric Adverse Event Rating scale (PAERS), for example (45) (Figure 2).

### 4.5 Limitations

This is a retrospective analysis. We only know the diagnoses of the inpatient treatment and the results of serum and urine analyses. Successful (outpatient) treatment that had taken place previously could have resulted in no diagnosis being made and could have led to wrong classification regarding on- or off-label use. We neither know the reasons for outpatient psychopharmacological therapy nor if the found drugs were still prescribed to our patients at admission. Moreover, the observed urine presence and/or serum levels could reflect misuse of antipsychotics (especially quetiapine or low-potential antipsychotics) and not the regular prescription (49, 50). However, the misuse of antipsychotics, for example, the use of quetiapine with alcohol, often results in intoxication. Patients with acute intoxications are not part of our sample. Off-label prescriptions might have had advantages over on-label medication that only prescribing doctors know (51). We cannot find out the dosages and dosage forms (long-acting vs. short-acting formulas, e.g., quetiapine levels of short and long-acting quetiapine differ), thus are not able to determine concentration-to-dose ratios. Serum concentrations and clinical outcomes may not be related (23, 26). GCMS was only a qualitative screening; the presence of aripiprazole was not detected. We only investigated serum concentrations of approved psychotropic substances (TDM); used off-label psychotropic drugs might have been in the therapeutic range, but please note that therapeutic drug levels are indication-specific. We could only analyze the presence of actual injuries on the skin or other parts of the body, we do not know if the found lesions were self-injuries, as ICD 10 does not allow classification of the intention of the injuries. There is the category MB23” Symptoms or signs involving appearance or behavior “in ICD 11; thus, it will be possible in the future to classify intentional self-injuries (52). The definition of polypharmacy normally includes a time criterion (e.g., > 30 or 60 days of concomitant use) and > 2 different psychotropic drugs. We were only able to prove the types of different drugs used at admission. We do not know if there was an outpatient psychotherapeutic treatment; cognitive behavioral therapy alone or in combination with antidepressants is effective in treating depressive disorders. Lastly, we examined only cases. Theoretically, re-admissions and repetitive TDM analyses were possible.

## 5 Conclusion

Suicidal adolescents use frequently prescribed psychotropic drugs at hospital admission. Off-label use, especially of antipsychotic agents, is common. The risk of supratherapeutic drug levels appears to be higher in suicidal female adolescents than in male adolescents. A critical discussion on the observed use of (sedating) psychotropic drugs is needed–particularly with regard to off-label-used drugs. The pre-pandemic prevalence rates of off-label use and supratherapeutic drug levels are a cause of concern in our adolescent population, as the pandemic might have led to higher prescription rates of psychotropic agents. At the beginning of inpatient treatment, ADRs should be excluded. After TDM and discussing other existing therapeutic options, there are possibilities for improvement of psychotropic drug therapy in both directions such as deprescribing and/or increasing existing drug therapy. TDM at the beginning of an inpatient treatment should be used as secondary prevention to enhance the safety and efficacy of the individual psychopharmacological treatment of adolescent patients. TDM at the beginning of inpatient therapy can provide useful information for the therapy of adolescents with mental disorders. Every inpatient treatment is an opportunity to find new treatment options (psychotherapy and psychotropic drug treatment) for suicidal adolescents with mental disorders. TDM should be routinely done in outpatient treatment as well. There is an urgent need for more real-world evidence on the effective treatment of adolescents with psychotropic drugs.

## Data availability statement

The raw data supporting the conclusions of this article will be made available by the authors, without undue reservation.

## Ethics statement

The studies involving humans were approved by Institutional review board Klinikum Nürnberg. The studies were conducted in accordance with the local legislation and institutional requirements. The human samples used in this study were acquired from samples that were routinely captured. Laboratory data was stored anonymized and separately from clinical data. Written informed consent for participation was not required from the participants or the participants’ legal guardians/next of kin in accordance with the national legislation and institutional requirements.

## Author contributions

IH, TB, and PN contributed to the study conception and design. IH wrote the first draft of the manuscript. All authors commented on previous versions of the manuscript and read and approved the final manuscript.
